# An Instantaneous Low-Cost Point-of-Care Anemia Detection Device

**DOI:** 10.3390/s150204564

**Published:** 2015-02-16

**Authors:** Jaime Punter-Villagrasa, Joan Cid, Cristina Páez-Avilés, Ivón Rodríguez-Villarreal, Esteve Juanola-Feliu, Jordi Colomer-Farrarons, Pere Ll. Miribel-Català

**Affiliations:** 1 Department of Electronics, University of Barcelona, Martí i Franquès 1, Barcelona 08028, Spain; E-Mails: cpaezaviles@el.ub.edu (C.P.-A.); ejuanola@el.ub.edu (E.J.-F.); jcolomer@el.ub.edu (J.C.-F.); pmiribel@el.ub.edu (P.L.M.-C.); 2 Department of Hemotherapy and Hemostasis, CDB, IDIBAPS, Hospital Clínic, Villarroel 170, Barcelona 08036, Spain; E-Mail: jcid@clinic.ub.es; 3 Centre de Recerca Matemàtica, Campus Bellaterra, UAB, Edifici C, Barcelona 08193, Spain; E-Mail: irodvill@crm.cat

**Keywords:** whole blood, hematocrit, impedance analysis, electronics, point-of-care

## Abstract

We present a small, compact and portable device for point-of-care instantaneous early detection of anemia. The method used is based on direct hematocrit measurement from whole blood samples by means of impedance analysis. This device consists of a custom electronic instrumentation and a plug-and-play disposable sensor. The designed electronics rely on straightforward standards for low power consumption, resulting in a robust and low consumption device making it completely mobile with a long battery life. Another approach could be powering the system based on other solutions like indoor solar cells, or applying energy-harvesting solutions in order to remove the batteries. The sensing system is based on a disposable low-cost label-free three gold electrode commercial sensor for 50 μL blood samples. The device capability for anemia detection has been validated through 24 blood samples, obtained from four hospitalized patients at Hospital Clínic. As a result, the response, effectiveness and robustness of the portable point-of-care device to detect anemia has been proved with an accuracy error of 2.83% and a mean coefficient of variation of 2.57% without any particular case above 5%.

## 1. Introduction

According to the World Health Organization (WHO), anemia is defined as a condition in which the number of red blood cells (RBCs) or their oxygen-carrying capacity is insufficient to meet physiological needs [1]. Anemia affects about two billion people, or 30% of the world's population. Pregnant women and children are the most vulnerable segment, and it is considered a worldwide health issue affecting both developed and developing countries [2,3]. The highest prevalence is in Africa (67.6%) and South-East Asia (65.5%). In the Eastern Mediterranean, the prevalence is 46% and around 20% in the other WHO regions, the Americas, Europe and Western Pacific [3]. The major health consequences in severe cases are pregnancy disorders, poor physical and cognitive development, and increased risk of morbidity, while less severe cases provoke weakness, fatigue and dizziness [3,4]. The most common cause of anemia is nutritional deficiencies, especially in developing countries due to severe malnutrition [5], or diseases like colon cancer or gastrointestinal lesions [6,7]. Other conditions causing anemia are inherited hematologic diseases such as sickle cell anemia or thalassemia causing hemolytic anemia [8,9], cancer treatments (chemotherapy and radiation) [10], and indirect causes, such as lower erythropoietin (EPO) production due to kidney disease [11]. Also, frequent blood donations may induce anemia in blood donors, especially females [12].

Evaluation of hemoglobin (Hb) concentration is the most reliable indicator for anemia detection; being a required condition in possible blood donors in most countries of the world, generically the only laboratory control test performed before donation [13]. Hematocrit (HTC) screening is also a reliable indicator for anemia, which is the proportion of blood volume occupied by RBCs, and is determined by cell number and size. HTC numbers below a certain reference range may indicate anemia or abnormal cell development [14]. Automated hematology analyzers provide the necessary information about HTC and Hb with a high degree of precision by means of a complete blood count (CBC), which represents an extremely useful tool for evaluating anemia [4]. However, hematology analyzers are huge and expensive devices which need to be operated by skilled technicians using venous blood sample, requiring a phlebotomy practiced by a specialist. Access to electricity to power the instrumentation is also required [15,16]. These different factors make the access to these devices very limited and often incompatible with the constraints of resource-limited settings [15]. Furthermore, access to this equipment is an issue in developing countries, where less reliable equipment and evaluation techniques are available [17]. These different factors push towards the development of point-of-care (PoC) anemia detecting devices that provide an easy to use, reliable and economic test for patients, the general public and first aid personnel screening for anemia. Detection of anemia with a short response time in a portable PoC device relying on a blood drop (50 μL sample that can be capillary collected [18]), provides reduced disposition decision time [19], improving patient satisfaction [20], and avoids inducing anemia or making it worse (phlebotomy is reported to induce anemia [21]).

Electrical impedance has been reported as an accurate solution for cellular detection, quantification and monitoring in different environments both *in-vivo* and *ex-vivo* [22–26]. Pop *et al.*, [24] presented a continuous *in-vivo* hematocrit monitoring in the human right atrium by venous catheter equipped with an impedance-measuring device. On the other hand, Pradhan *et al.*, [25] studied the electrical properties of blood and its constituents using Electrochemical Impedance Spectroscopy (EIS). Ramaswamy *et al.*, [26] performed a blood coagulation test based on a custom microfluidic device and the electrical impedance detection of whole blood samples. Our group has previously studied this technique and has used it in the design of a novel anemia detection device [27]. The device presented reliable, sensitive and robust hematocrit detection, relying on low-cost straightforward electronic equipment and sensing systems. Moreover, the impedance measurement technique provided an actual hematocrit instantaneous measurement, with a wide measuring range oscillating from 0% to 100%, while other techniques, such as optical photometry, a slower hemoglobin measurement for a subsequent hematocrit indirect calculation. Additionally, considering a truly PoC device, the impedance measurement technique is a much less complex technique that does not require any optical measurement or chemical agent, resulting in a more economic, longer battery life and environmentally safe device.

This work presents an improvement on the previously reported device [27], as a compact, economic and portable PoC solution, for instantaneous detection of hematocrit through whole blood samples. The previously developed device relied on full spectrum analysis of blood samples by means of a Digital Lock-In Amplifier (DLIA) based on a Frequency Response Analyzer (FRA) approach. Full spectrum analysis involved a microprocessor for system control and data processing, and further electronics for signal conditioning, such as an SPI controlled oscillator AD9833 from Analog Devices (Norwood, MA, USA), and a 12-bit dual ADC ADC12D040 from Texas Instruments (Dallas, TX, USA) capable of converting simultaneously two analogue input signals at 40 MSPS. Moreover, a real-time platform sbRIO9632 from National Instruments (Austin, TX, USA) was used for fast software prototype development and versatility. All these electronics were a major drawback in terms of power consumption, size and price, when aiming for a specific PoC device.

The system is composed of an economic and reusable low-cost electronic device and a plug-and-play disposable commercial sensor. This sensor is based on three screen-printed electrodes for an envisaged sample of 50 μL. In order to validate the instantaneous hematocrit detection system, different blood samples, which came from the manipulation and dilution of whole blood samples extracted from 4 healthy donors, have been studied. These samples were randomly obtained from hospitalized patients of Hospital Clínic located in Barcelona, Spain.

## 2. Materials and Methods

### 2.1. Hematocrit, Electrodes, Impedance Measurement and Sensing System

A typical cellular electrical model for dilute cell suspensions can be described as a network of electrical passive components, so the biological electrical impedance is the response to applying an electrical stimulus to a biological material through a sensing system and measuring its electrical response as defined by Ohm's law [28] (Equation (1)):

(1) $$Z_{\text{CELL}}=\frac{V_{\text{CELL}}}{I_{\text{CELL}}}$$

In this work we have adopted a configuration of three electrodes, which are defined as follows: the *working electrode* (WE), where electrical response of the object under investigation is measured; the *reference electrode* (RE), which tracks the electric signal and the *counter or auxiliary electrode* (CE), which supplies the current required. With this electrode configuration, the problematic behavior of the simpler two electrodes topology is avoided [27]. This configuration is defined by the WE, where the sample is placed and the electrical signal is applied, and the CE, which tracks the solution potential and supplies the current required for experience, creating a polarization effect causing a distortion of the applied electrical signal. With this sensing system topology, the electrical stimulus applied is an ac voltage (V_CELL_), while the electrical response is the current flow through the WE electrode (I_CELL_).

**Figure 1.**
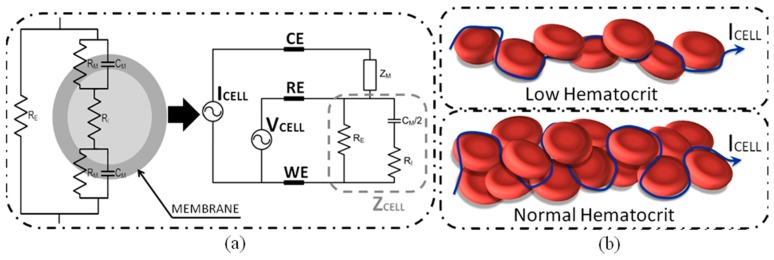
(**a**) Red Blood Cell (RBC) electrical model. Three electrodes topology for RBC sample; (**b**) Current flow path through different blood samples with different hematocrit. (Reproduced from [27] with kind permission from IEEE Publishers).

Hence, electrical properties can be described as a passive electrical components network [29] (Figure 1a), so the current related to the electrical stimulus (I_CELL_) can flow through an external cellular path (R_E_) or across the cell membrane (RM∥CM) and go through the intra-cellular medium (R_I_). Hill and Thompson [30] confirmed the close relation between hematocrit and impedance at low frequencies (up to 100 kHz), where I_CELL_ flow path is located outside the RBCs across RE impedance. This phenomenon implies a correlation of impedance and hematocrit, so that an increment in RBCs makes the current flow path larger between the reference and working electrodes, becoming an increment on Z_CELL_ impedance due to an increment of R_E_ impedance (Figure 1b).

This phenomenon has been studied previously [27], demonstrating the feasibility of the impedance measurement technique to perform an easy, fast and sensitive hematocrit detection, evaluated through comparison with complete blood count (CBC) by using a clinical hematology analyzer, the Advia 2120 from Siemens AG (Munich, Germany). This sensing system is a low-cost disposable three-electrode sensor that works with 50 μL blood samples. This is the standard volume for a whole blood drop, which is easy to manipulate by clinical laboratory technicians using standard clinical laboratory tools. Furthermore, the sensor electrodes must be made of gold, an acknowledged bio-compatible material. Different commercial sensors have been evaluated, such as AC1 sensor from BVT Technologies (Brno, Czech Republic), or the G-AUG sensor series from Bio-Logic SAS (Claix, France). The commercial sensor that best reaches the defined specifications is the C223AT from Dropsens (Llanera, Spain). This sensor is specifically designed to work with 50 μL drop samples and has gold screen-printed electrodes of 1.6 mm diameter. Blood samples were easily put on top of the sensor electrodes with an automatic pipette.

### 2.2. Blood Samples

Four blood samples were obtained in 4-mL tubes containing ethylenediaminetetraacetic acid (EDTA 7.2 mg from BD Vacutainer, (Franklin Lakes, NJ, USA) from four random hospitalized patients in Hospital Clinic. To obtain a larger sample collection, the initial four whole blood samples were centrifuged (Jouan CR412 from DJB Labcare, Newport Pagnell, UK) at 2200 rpm for 15 min in order to separate blood plasma from RBCs. Finally, 24 blood samples were obtained diluting obtained RBCs in different volumes of blood plasma using a Labopette Manual 10–100 μL automatic pipette from Hirschmann Laborgeräte (Louisville, KY, USA). We performed a complete blood count (CBC) of the 24 blood samples with an ABX Micros 60 haematology analyzer (Horiba, Kyoto, Japan) which reported the hemoglobin and hematocrit results as g/dL and percentage (%), respectively. This analyzer used electrical impedance technology to perform the CBC. With this methodology, whole blood is aspirated into the system, the sample stream is split, one portion is used for hemoglobinometry and one portion is used for RBC counting and size. Hemoglobinometry is based on RBC analysis and measurement of hemoglobin concentration by absorbance of spectrophotometry. RBC counting and size analyses are performed by passing the RBCs singly through a small direct current. The temporary increase in impedance caused by the passage of the cell provides information about RBC number and RBC volume. Hematocrit is calculated from the measured hemoglobin, RBC number and RBC volume [31]. Therefore, the obtained hematocrit (HCT (%)) and hemoglobin (Hb (g/dL)) for the different blood samples are shown in Table 1.

### 2.3. System Description

**Figure 2.**
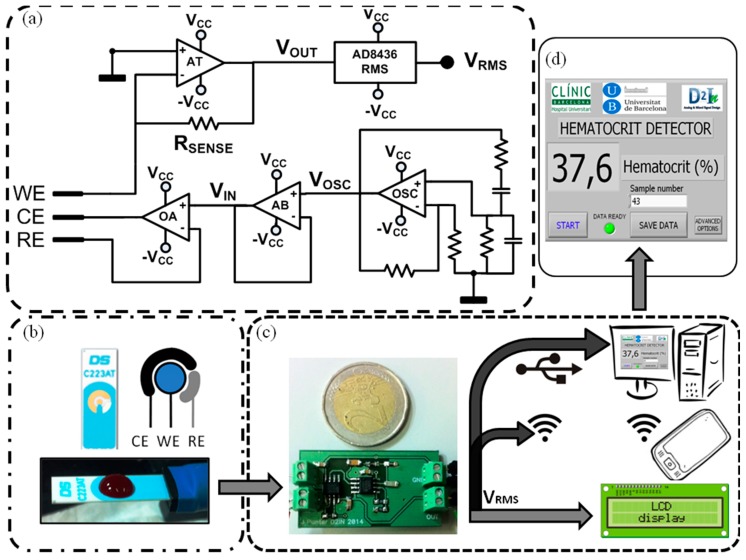
(**a**) Custom electronic instrumentation; (**b**) Commercial C223AT disposable sensor with a 50 μL whole blood drop; (**c**) Device prototype electronics and different suitable and functional user readout interfaces (the reference coin has a diameter of 25.75 mm); (**d**) Actual user-friendly front-end user interface develop with Labview^©^. (Reproduced from [27] with kind permission from IEEE Publishers).

A full custom electronic circuit was specifically designed to carry out impedance measurements using a disposable three-electrode C223AT sensor. The electronic system was designed based on the specifications found in the study previously reported [27]. The architecture of the device is divided in three parts: an oscillator that provides the ac voltage signal (V_CELL_), a sensor driving instrumentation and a rms-to-dc converter (Figure 2a). The oscillator is a Wien bridge oscillator, a stable output amplitude with low distortion. The operational amplifier (OSC in Figure 2a) is the AD8066 from Analog Devices, which is a low-cost, high speed Junction Gate Field Effect Transistor (JFET) amplifier dual supply with low leakage current and distortion, in order to provide a stable sinus voltage signal with low offset (V_OSC_).

The oscillator has been configured to provide a voltage sinus signal of 33 kHz, a well-suited frequency for hematocrit detection using the C223AT [28]. Moreover, as the AD8066 commercial integrated circuit provides two isolated amplifiers, the second amplifier has been used as a voltage follower (AB in Figure 2a), due to its high speed and low distortion specifications for isolating the oscillator from the potentiostat. The sensor driving instrumentation is based on a potentiostat with a transimpedance amplifier current readout stage [32], composed of an operational amplifier to bias the sensor and an operational amplifier in transimpedance configuration as a sensing system current readout. The operational amplifier (OA in Figure 2a) is the AD8066 from Analog Devices, which is perfectly designed for singly driving the electrodes and track the voltage-biasing signal (V_IN_) to the electrodes (2).

The JFET high input impedance avoids RE electrode voltage distortion, considering the low load impedance on the sensing system [27], and the high bandwidth and slew-rate provides stability to the system. The second amplifier included on the integrated circuit package has been configured as the transimpedance amplifier (AT in Figure 2a). The transimpedance amplifier converts the current through the electrodes (3) into a voltage signal (V_OUT_) by means of a sensing resistor (R_SENSE_ in Figure 2a). The main drawback of this configuration, an amplifier with low input impedance [capitol intech antic], is avoided with the JFET input of the AD8066:

(2) $$Z_{\text{CELL}}=\frac{V_{\text{IN}}}{I_{\text{CELL}}}$$

(3) $$V_{\text{OUT}}=-R_{\text{SENSE}}\;\cdot \;I_{\text{CELL}}=-R_{\text{SENSE}}\frac{V_{\text{IN}}}{Z_{\text{CELL}}}$$

Finally, the rms-to-dc converter is the AD8436 from Analog Devices, which computes a precise dc equivalent (V_RMS_) of the transimpedance amplifier ac signal (V_OUT_). It is a low cost, low power device, with wide dynamic input range and wide bandwidth that provides low distortion with a Zero subthreshold swing Field Effect Transistor (ZFET) input buffer for electronic isolation from the instrumentation stage. Considering that the electrodes voltage biasing signal (V_IN_) and the sensing resistor (R_SENSE_) are stable and well known, the rms dc variations of V_OUT_ are only related to the variations of Z_CELL_. The device dc output voltage (V_RMS_) is inverse compared to the hematocrit values, so as the hematocrit increases, V_RMS_ decreases. The device usage is very simple not requiring any qualified users. A blood sample is placed on top of the sensor electrodes and, once the power supply is connected, the device dc output voltage (V_RMS_) is ready for reading on the output pin. On the presented manuscript, a software interface on an external computer with Labview^©^, from National Instruments, controlled an electric switch to enable the power supply (an external source at ±5 V), and presents the resultant data on a user-friendly user interface (Figure 2d). The electronics and the computer were connected by means of a NI USB-6361 data acquisition (DAQ) device from National Instruments. However, the presented device readout stage can be greatly improved to address different applications and user skills (Figure 2c), such as an integrated Liquid Crystal Display (LCD) for an untrained user self-screening, a remote computer connected to the electronics by means of an standardized protocol (USB, ethernet, *etc.*), or a wireless communication protocol for self-monitoring device in telemedicine applications. Additionally, the presence of an electrical signal directly correlated to hematocrit allows the device implementation as a controller of other clinical actuators in different environments and situations increasing functionality.

The overall low cost and low power system composed of optimized straightforward standards for instrumentation electronics, results in a reusable, robust and low consumption device (300 mWh) making it completely mobile with a long battery life time. Moreover, it is important to highlight that it is an easy to manipulate and economic electronics (less than 10 € per device), providing an instantaneous impedance measurement.

## 3. Results

**Figure 3.**
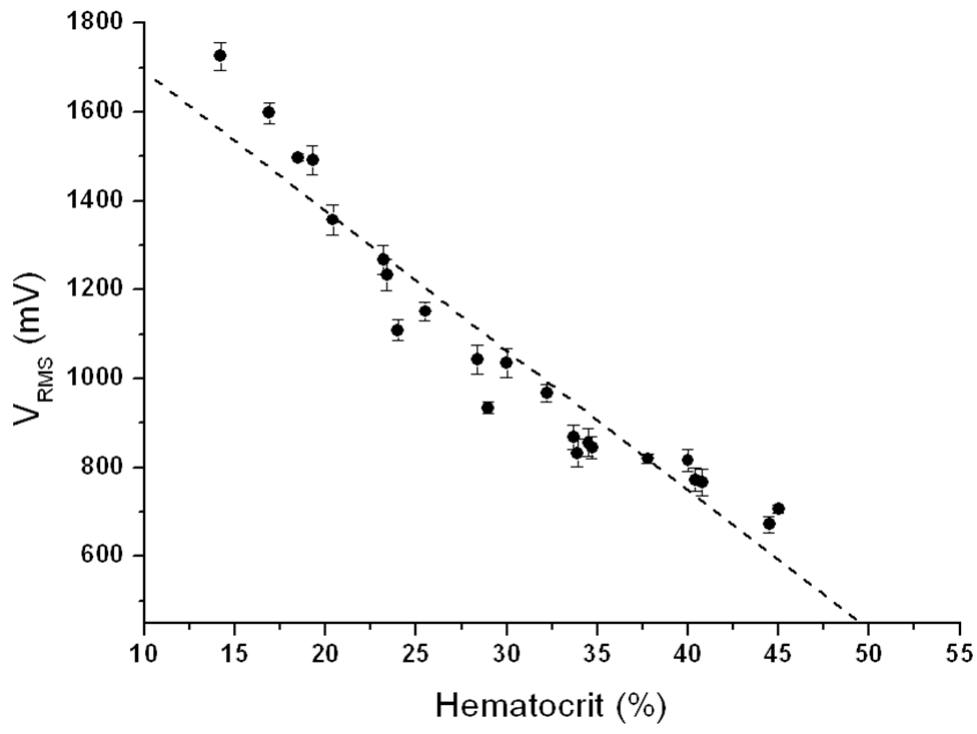
Measured output dc voltage (V_RMS_ (mV)) mean value (*n* = 5) as a function of blood samples hematocrit (Hematocrit (%)).

A small, compact and portable device for point-of-care early instantaneous detection of anemia was prototyped. For its validation, we analysed 24 consecutive blood samples from patients hospitalized at Hospital Clínic in Barcelona. We performed a complete blood count (CBC) of the blood samples with a haematology analyser, the Advia 2120 from Siemens AG, which reported the hemoglobin and hematocrit results as g/dL and percentage (%), respectively. We tested all samples with the prototype within 2 h of blood collection and CBC. As it is an instantaneous detector with a time response of several milliseconds, to evaluate system precision and accuracy, every whole blood sample was tested 5 times consecutively using fresh sensors and fresh sub-samples. Figure 3 depicts the output dc voltage (V_RMS_) of the device and compares it with the different whole blood samples hematocrit (Hematocrit (%)).

We used the Linear Regression (LR) analysis to measure the Pearson's correlation coefficient (r) and coefficient of determination (r^2^) between the reference method (CBC method) and the custom electronic device method, where the output voltage (V_RMS_) mean value (*n* = 5) has been compared with whole blood samples CBC hematocrit. The LR slope (β) defines the sensitivity, in terms of mV per hematocrit percentage (mV/%). Meanwhile the hematocrit detection accuracy (%) is the relation (4) between r^2^ and β:

(4) $$V_{\text{RMS}}(mV)=\alpha +\beta \;\cdot \;\text{Hematocrit}(\%)$$

In Table 2 the experimental results of whole blood samples hematocrit (HCT (%)), the output voltage mean value (V_RMS_ (mV)) of the five measurements performed with each whole blood sample and its standard deviation (SD (mV)) are shown. Precision was evaluated with the coefficient of variation: the standard deviation (SD (mV)) divided by the mean value (V_RMS_ (mV).

The proposed anemia detector device presented great accuracy at detecting hematocrit, with a Pearson's correlation coefficient of −0.96, an accuracy error of 2.83% hematocrit, and a coefficient of determination of 92.72%. The mean coefficient of variation is 2.57% without any particular case above 5%. Acceptable values in quality control procedures in clinical haematology measurements show a coefficient of variation less than 5% [30]. The device presents reliable, sensitive and robust anemia detection compared with other commercial PoC devices for anemia detection, such as AnemiaCheck from Express Diagnostics (Blue Earth, MN, USA), STAT-Site from Stanbio Laboratory (Boerne, TX, USA), or HemoPoint H2 from Alere (Waltham, MA, USA), where its detection performance is similar to the proposed prototype but with much slower response.

Recently, other PoC anemia devices have been published, such as a color-based diagnostic test for self-screening/self-monitoring of anemia presented by Tybursky *et al.*, [33], a novel PoC diagnostic test for self-screening, self-monitoring of anemia. The device measures hemoglobin (Hgb) levels, which are estimated via visual interpretation by the user using a color scale. This system presents several performance drawbacks when compared with our device. First of all, the readout stage, based on a color scale, relies on the visual interpretation of the user, which could introduce errors on the Hgb levels data interpretation, and reduces considerably the system resolution. Furthermore, the principle of operation of the color-based POC device is based on biochemical reactions, where the blood comes into contact with a reagent solution initiating a redox reaction, which is a slow and destructive procedure.

## 4. Market and Technology Transfer Challenges

Commercialization of biosensors technology is still delayed compared with research in academia. This reduced technology transfer activity could be attributed to technical barriers or cost considerations. Therefore, devices must be versatile to allow automation at a competitive cost [34]. Additionally, to ensure success in the development, innovation and technology transfer, it is necessary to foster a particular scenario typified by the convergence of technologies and disciplines [35]. In this context, one of the main characteristics of the proposed device is its multidisciplinarity: in an effort to integrate knowledge from various dimensions, main actors and activities. The cross-disciplinary interaction must be examined in the way scientific knowledge flows between engineers, researchers and physicians. Currently, there are huge opportunities to be exploited by researchers and innovation managers in the development of high-tech products, above all in the field of medical devices. As such, the University–Hospital–Industry–Administration (plus Citizens) system should emerge as an essential five-helix leading to a successful technology transfer and commercialization of public-funded medical devices [36].

In biomedical research, there is a great need for multipurpose and reliable telemetric tools. By using sensor inputs, such devices allow the automated gathering of information on physiological parameters minimizing adverse effects for the patients [35]. In this context the proposed device could improve the diagnosis especially in countries where clinics are often many miles away from villages, where there is absence of laboratory facilities and trained staff, or there are hostile environmental conditions [15]. Additionally, this device could overcome some of the complications related to blood extractions used in conventional methods, including hematoma formations, nerve damage, pain, hemoconcentration, extra-vasation, iatrogenic anemia, arterial puncture, petechiaes, allergies, infection, syncope and fainting, excessive bleeding, edema or thrombus [37].

PoC testing promotes a shift away from traditional diagnostic tests in the clinical laboratory setting to near-patient situations, improving the timely diagnostic information so as to make informed decisions regarding diagnosis and treatment. Rapid advances are already being achieved at remarkably low cost with modest investments; therefore there is a high growth rate market [38]. PoC devices represent 31% of the diagnostics market, 18% glucose testing, 11% professional PoC products, and 2% over-the-counter [39]. It is expected that the global market will increase to US$16.5 billion by 2016 and $34.6 billion by 2021 [40]. Additionally, the total LoC-based biochip market was US$2.4 billion in 2009 and was projected to increase to US$5.9 billion in 2014. This should be a powerful incentive for commercial efforts to move toward true global health solutions [15].

## 5. Conclusions

In this work, a novel point-of-care (PoC) device for instantaneous anemia detection based on custom instrumentation electronics, impedance measurement technique and a disposable low-cost sensor has been designed, fabricated and tested. The device has been proved to exhibit reliable, robust and effective results using disposable commercial sensors with 50 μL whole blood samples. Advantages of the proposed device include: (i) the facility of use; (ii) compactness and small size; (iii) portability; (iv) less invasive and less quantity of blood; (v) rapidity and accuracy of results; and (vi) low-cost accessibility. These characteristics are valuable for anemia-risk patients, especially for pregnant women, neo-nates, pediatric patients and elderly, but also for chronically anemic patients, such as cancer patients receiving chemotherapy, patients with renal failure, patients with chronic inflammatory/immunologic disorders, or patients with primary hematologic disorders. Low manufacturing cost and the accessible price are important advantages, especially in disadvantaged regions where the health domain is undervalued. Commercial devices for PoC anemia detection, based on microfluidic manipulator devices, such as AnemiaCheck from Express Diagnostics, or on photometry hemoglobin detection, such as STAT-Site from Stanbio Laboratory, or HemoPoint H2 from Alere, relay on slower hemoglobin measurement for a subsequent hematocrit indirect calculation, with results open to user interpretation as the readout stages are optically represented. The presented device outputs instantaneous reliable results based on electric voltage data directly correlated to hematocrit, the system have further versatility in terms of applications compared with other commercial devices, such as a point-of-care hematocrit detector, a monitoring device for telemedicine applications, or as a controller of other clinical actuators. Furthermore, unlike actual clinical equipment for blood analysis, whole blood samples are not destroyed in the measurement process and the adverse effects for patients and blood samples are being mitigated.

PoC anemia diagnostic devices recently published, such as a color-based diagnostic test by Tybursky *et al.*, [40], present severalby of the user, which could introduce errors on the data interpretation, and reduces the system resolution, being a slower and destructive technique, with less functionalities in Point-of-Care and Lab-on-a-Chip devices for medical and research applications.

Twenty four blood samples, obtained from four patients hospitalized at Hospital Clínic, were used to demonstrate the feasibility of the impedance measurement technique to perform an easy, fast and low-cost hematocrit study using disposable commercial sensors. The system has been evaluated through comparison with complete blood count (CBC) using a clinical haematology analyser. The anemia detection device has a Pearson's correlation coefficient of −0.96 and a coefficient of determination of 92.72% hematocrit. Coefficient of variation is below 5%, with a worst-case accuracy error of 2.83%. Additionally, as the system is based on straightforward low cost and low power standards on instrumentation electronics and sensing, it represents an economic, portable, safe and reliable system of anemia detection with a high degree of integration for the clinical environment, driving the development of a truly autonomous, portable and versatile device relying on the presented work. In Table 3 is presented a performance comparison table with different commercial and published anemia PoC diagnostic test.

Once again, this demonstrates the importance of a multidisciplinary team that integrates the clinical research with the university, in an effort to obtain a cross-fertilization development that aims to satisfy medical but also social needs through R + D + i. With this combined effort and symbiosis, it is possible to obtain innovative products and also reduce the time-to-market in medical research settings.

In summary, this paper describes the design, development and test of a novel instantaneous anemia detection PoC device with low cost disposable commercial sensors and instrumentation electronics. The device presents reliable, sensitive and robust anemia detection, relying on low power straightforward electronic equipment and sensing systems for hematocrit monitoring.

## Figures and Tables

**Table 1.** Twenty four blood samples obtained in Hospital Clínic from four random hospitalized patients. Blood samples were obtained diluting RBCs in different volumes of blood plasma. Hematocrit (HCT (%)) and hemoglobin (Hb (g/dL)) values for the different blood samples were obtained by means of a complete blood count (CBC).

**HCT (%)**	**Hb (g/dL)**	**HCT (%)**	**Hb (g/dL)**	**HCT (%)**	**Hb (g/dL)**
14.2	5.0	25.5	9.0	34.7	12.4
18.5	6.7	28.4	9.7	37.8	12.5
18.9	6.6	29.0	9.8	40.0	12.8
19.3	7.0	29.2	9.7	40.4	14.0
20.4	6.9	30.0	10.5	40.8	14.0
23.2	7.8	33.7	11.5	44.5	14.9
23.4	8.1	33.9	11.8	45.0	15.2
24.0	8.1	34.5	11.5	50.6	18.0

**Table 2.** Device validation with whole blood samples.

**HCT (%)**	**V_RMS_ (mV)**	**SD (mV)**	**HCT (%)**	**V_RMS_ (mV)**	**SD (mV)**	**HCT (%)**	**V_RMS_ (mV)**	**SD (mV)**
14.2	1725.92337	31.28	25.5	1152.10028	21.24	34.7	845.70887	25.20
16.9	1598.31218	24.08	28.4	1043.23605	32.28	37.8	820.04855	10.32
18.5	1498.75603	8.12	29.0	934.82407	13.08	40.0	816.04674	25.08
19.3	1491.64678	32.4	30.0	1035.4325	33.88	40.4	773.16798	25.20
20.4	1358.14206	34.56	32.2	968.69188	20.04	40.8	766.09567	30.36
23.2	1268.13432	33.00	33.7	869.14198	26.44	44.5	671.69091	18.52
23.4	1233.56278	34.16	33.9	832.33453	31.32	45.0	707.49377	8.76
24.0	1109.21312	23.28	34.5	856.91271	30.12	50.6	490.1229	6.32

**Table 3.** Comparison table with different commercial and published anemia PoC diagnostic test.

**Device**	**Test Time (s)**	**Range (HCT (%) and Hb (g/dL))**	**Standard Deviation (%)**	**Coefficient Variation (%)**
Presented device.	Instantaneous	HCT: 0%–100%	2.83%	2.57%
STAT-Site.	900	HCT: 12%–42%	0.74%	4.10%
Alere HemoPoint H2 System.	120	Hb: 5.6 g/dL–20.6 g/dL	NA	4.20%
Anemia Check.	60	Hb: 0 g/dL–25.6 g/dL HCT: 36%–54%	NA	1.5%
Tybursky *et al.*, (2014) [40]	60	Hb: <9 g/dL–>12 g/dL	NA	NA
